# Induced Pluripotent Stem Cell–Derived Hepatocytes From Patients With Metabolic Dysfunction–Associated Steatotic Liver Disease Exhibit Early Mitochondrial Dysfunction

**DOI:** 10.1016/j.jcmgh.2026.101853

**Published:** 2026-08-13

**Authors:** Dounia Le Guillou, Kevin Siao, Chris L. Her, Caroline C. Duwaerts, Jacquelyn J. Maher

**Affiliations:** Department of Medicine, University of California San Francisco, San Francisco, California; The Liver Center, University of California San Francisco, San Francisco, California

Mitochondria are central to cellular energy production and homeostasis. In metabolic dysfunction–associated steatotic liver disease (MASLD), hepatocellular lipid accumulation triggers mitochondrial stress by increasing the demand for fatty acid oxidation (FAO).^1^ In early MASLD, mitochondria adapt to lipid accumulation by raising their oxidative capacity and activating antioxidant defenses^2^, ^3^, ^4^, ^5^; however, as metabolic dysfunction–associated steatotic liver transitions toward metabolic dysfunction–associated steatohepatitis (MASH), this adaptation deteriorates,^6^ resulting in the accumulation of harmful reactive oxygen species (ROS). Excess mitochondrial ROS promote lipid peroxidation and activate apoptotic and inflammatory signaling pathways.^7^ Moreover, ROS can impair mitochondrial biogenesis, resulting in a reduction of functioning mitochondrial mass. These observations underscore the importance of mitochondrial ROS as drivers of MASLD and MASH.

Our group uses induced pluripotent stem cell–derived hepatocytes (iPSC-Heps) to model human MASLD. We recently reported that iPSC-Heps from patients with biopsy-proven MASLD spontaneously develop steatosis and display a transcriptomic signature distinct from healthy subjects.^8^ In this study, we evaluated mitochondrial function in patient-derived MASLD iPSC-Heps to determine whether they recapitulate the mitochondrial disturbances observed in MASLD livers in vivo.

We selected induced pluripotent stem cell (iPSC) lines from 10 patients with MASLD and 10 healthy control subjects whose transcriptomic profiles after differentiation to iPSC-Heps clearly placed them into distinct groups.^8^ The demographic features of the iPSC donors are shown in Supplementary Table 1. Following differentiation of iPSCs to iPSC-Heps, MASLD iPSCs displayed spontaneous triglyceride (TG) accumulation, whereas control iPSC-Heps did not (Figure 1*A* and *B*). There was no difference in mitochondrial mass (Figure 1*C*) between MASLD and control iPSC-Heps, nor was there any difference between the 2 groups in the activity of citrate synthase or the expression of genes related to mitochondrial biogenesis (Supplementary Figure 1*A* and *B*). We evaluated mitochondrial oxygen consumption rates (OCRs) and found that MASLD, but not control, iPSC-Heps significantly increased their maximal OCR when cultured with galactose to force the use of oxidative metabolism for energy production (Figure 1*D*). The change in OCR between glucose and galactose conditions correlated positively with intracellular TG content (Figure 1*E*) and was associated with the reduced expression of genes in oxidative phosphorylation (OXPHOS) complexes II and IV (Supplementary Figure 1C).

**Figure 1:**
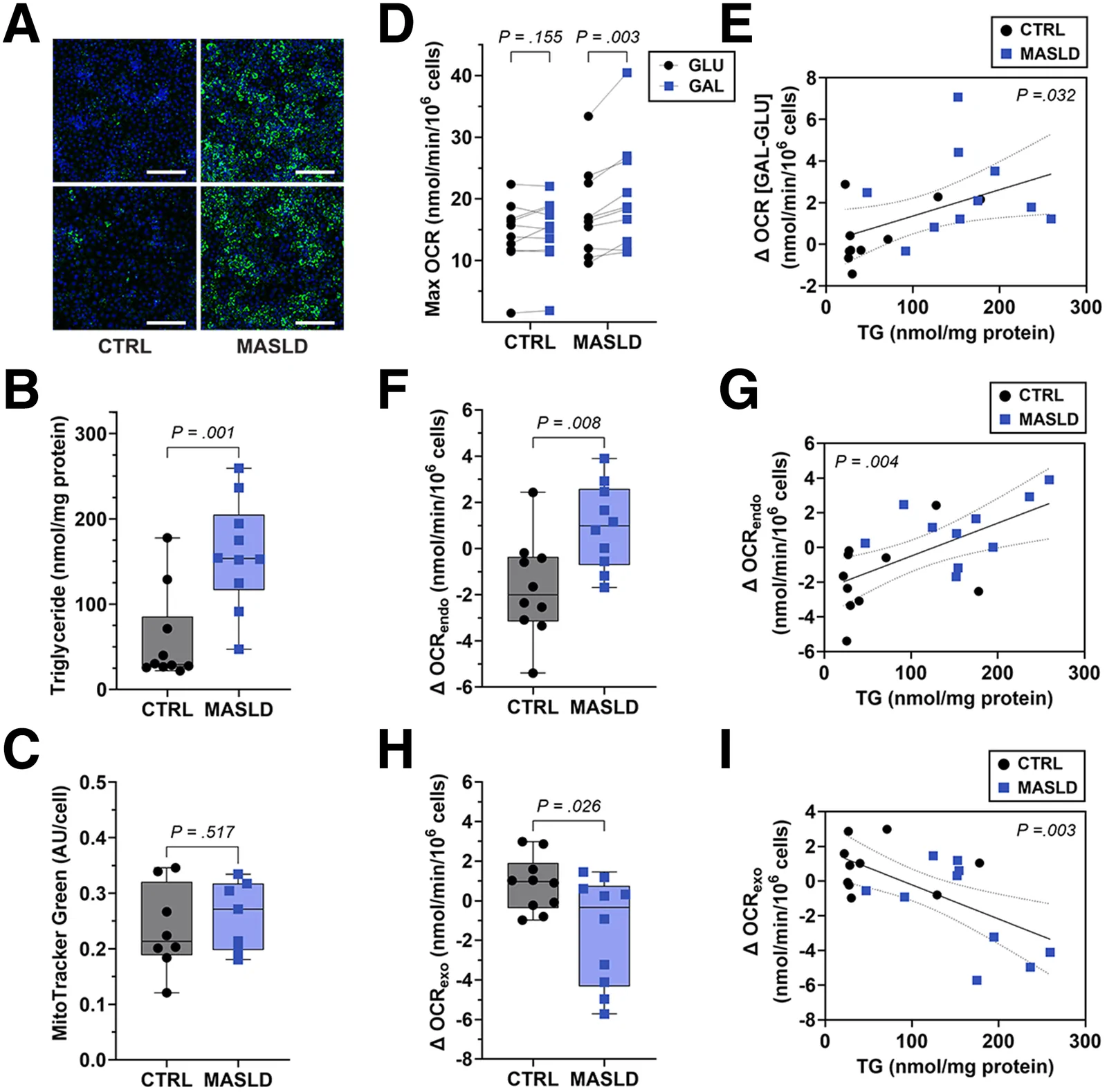
**Lipid accumulation and mitochondrial function in metabolic dysfunction–associated steatotic liver disease (MASLD) vs control (CTRL) induced pluripotent stem cell–derived hepatocytes (iPSC-Heps).** (*A*) Lipid droplets in 2 MASLD and 2 CTRL iPSC-Heps stained with BODIPY 493/503. Scale bar, 200 μm. (*B*) Cellular triglyceride (TG) content. (*C*) Mitochondrial mass based on MitoTracker Green staining (N = 8 CTRL and 7 MASLD). (*D* and *E*) Maximal oxygen consumption rate (OCR) in glucose (GLU) or galactose (GAL) and correlation between Δ OCR [GAL-GLU] and TG content. (*F* and *G*) Maximal endogenous fatty acid oxidation (FAO)-linked OCR and correlation with TG content. (*H* and *I*) Maximal exogenous FAO-linked OCR and correlation with TG content. Unless otherwise indicated, N = 10 CTRL and N = 10 MASLD; each symbol represents 1 subject. *P* values in (*B, C, F* and *H*) represent unpaired and (*D*) paired Student *t* tests. *P* values in (*E, G* and *I*) represent F-tests; *dotted lines* represent 95% confidence intervals.

To determine whether FAO is dysregulated in MASLD vs control iPSC-Heps, we measured FAO-linked OCR in the setting of endogenous and exogenous fatty acid. Endogenous FAO-linked OCR was higher in MASLD than control iPSC-Heps (Figure 1*F*) and correlated positively with iPSC-Hep TG content (Figure 1*G*). This was true despite the downregulation of several genes involved in fatty acid degradation (Supplementary Figure 1D). In contrast, when iPSC-Heps were challenged with exogenous palmitate, FAO-linked OCR was significantly lower in MASLD than control iPSC-Heps (Figure 1*H*) and correlated negatively with cellular TG content (Figure 1*I*). The latter result suggests that MASLD iPSC-Heps, which already contain excess lipid, are unable to accommodate an exogenous lipid challenge, signaling mitochondrial dysfunction. MASLD iPSC-Heps also produced significantly more ROS and less adenosine triphosphate than control iPSC-Heps when challenged with palmitate (Figure 2*A*; Supplementary Figure 1F), despite an increase in the expression of several genes involved in antioxidant defense (Supplementary Figure 1E).

When viewed in aggregate, our functional studies indicate that iPSC-Heps from patients with MASLD display disturbances in mitochondrial respiration resembling those in the intact human liver during the early stages of MASLD.^3^ Specifically, MASLD iPSC-Heps have a low threshold for utilizing endogenous fatty acids for energy production but have difficulty coping with an exogenous fatty acid load, resulting in ROS production and reduced adenosine triphosphate generation. This mimics findings in rodents^9^ and humans^2^^,^^3^^,^^5^ in which there is an early increase in mitochondrial respiration during the development of MASLD that is then lost in MASH.

Genetic risk has been implicated in the occurrence of hepatic mitochondrial alterations in humans with MASLD.^10^ We evaluated genetic risk in our cohort by calculating a weighted polygenic risk score (PRS) for each subject. Although there was a trend toward higher PRS in the MASLD group, it did not reach statistical significance (Figure 2*B*). When we assessed the metabolic phenotypes of iPSC-Heps as a function of PRS, we found no correlation (Figure 2*C–F*). These findings suggest that genetic background, at least with respect to the known single nucleotide polymorphisms tested, is not the sole determinant of MASLD-related metabolic dysfunction in iPSC-Heps.

**Figure 2:**
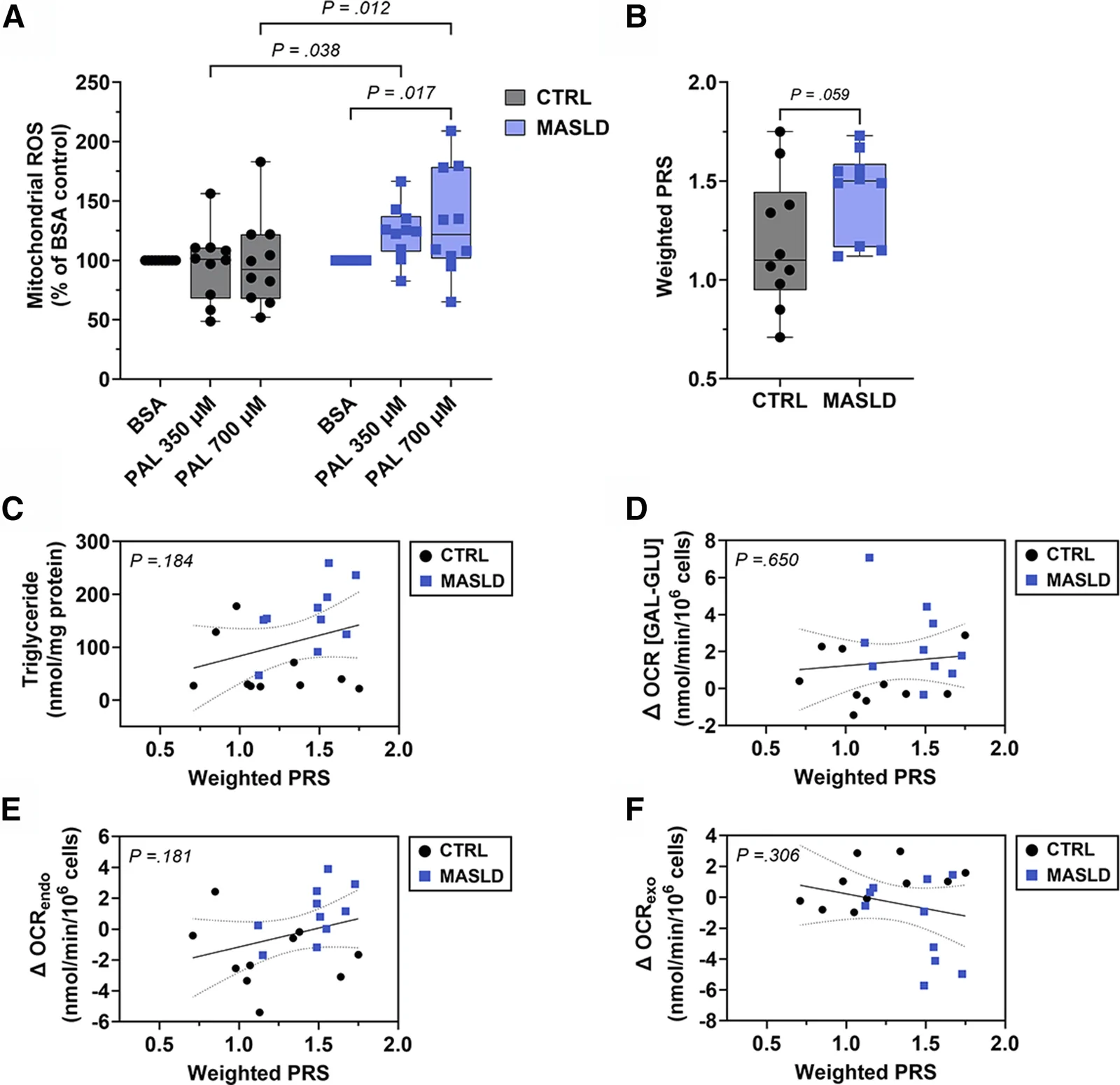
**Mitochondrial reactive oxygen species (ROS) production in induced pluripotent stem cell–derived hepatocytes (iPSC-Heps); the impact of polygenic risk on the iPSC-Hep phenotype.** (*A*) Mitochondrial ROS production in iPSC-Heps treated with bovine serum albumin (BSA) or palmitate (PAL). *P* values from 2-way analysis of variance with Šídák’s post hoc testing. (*B*) Weighted polygenic risk score (PRS) in control (CTRL) and metabolic dysfunction–associated steatotic liver disease (MASLD) iPSCs. *P* value from unpaired Student *t* test. (*C–F*) Correlations between weighted PRS and triglyceride (TG) content (*C*), mitochondrial oxygen consumption rate (OCR) (*D*), and endogenous (*E*) and exogenous (*F*) fatty acid oxidation (FAO). *P* values from F-tests; *dotted* lines represent 95% confidence intervals. N = 10 CTRL and 10 MASLD; each symbol represents 1 subject. GAL, galactose; GLU, glucose.

A key strength of our study is that we reproduced features of human MASLD in patient-derived iPSC-Heps that heretofore could only be studied in liver tissue. Moreover, we demonstrated that mitochondrial alterations are detectable in iPSC-Heps from several unrelated donors with MASLD, underscoring the utility of population-based iPSC modeling to investigate disease pathogenesis. A limitation of the study is the small number of subjects in each group.

In summary, iPSC-Heps from patients with biopsy-proven MASLD exhibit mitochondrial alterations representative of early MASLD in vivo. Several of these alterations arise even without an exogenous metabolic challenge, indicating that MASLD iPSC-Heps are intrinsically susceptible to metabolic stress. Importantly, mitochondrial dysfunction in MASLD iPSC-Heps does not correlate directly with genetic risk. This is seemingly at odds with human data in vivo^10^ and warrants further investigation.

## Supplementary Material

Supplementary Material

Extended PDF
