# Supplementary material for: Induced Pluripotent Stem Cell–Derived Hepatocytes From Patients With Metabolic Dysfunction–Associated Steatotic Liver Disease Exhibit Early Mitochondrial Dysfunction
Source: Cell Mol Gastroenterol Hepatol. 2026 Aug 13;21(1):101853. doi: 10.1016/j.jcmgh.2026.101853 (PMC13677320; doi:10.1016/j.jcmgh.2026.101853)
Supplement: Supplementary file 1 [file mmc1.pdf]

Supplemental Information for

Induced pluripotent stem cell-derived hepatocytes from patients with MASLD exhibit early mitochondrial dysfunction

Dounia Le Guillou,[1,2] Kevin Siao,[1,2] Chris L. Her,[1,2] Caroline C. Duwaerts[1,2] and Jacquelyn J. Maher[1,2]

[1]Department of Medicine and [2]Liver Center, University of California, San Francisco

METHODS

Generation of iPSCs and iPSC-Heps. Peripheral blood mononuclear cells were used to generate iPSCs from all subjects. iPSCs were reprogrammed by Cellular Dynamics International, Inc. (Novato, CA) using a virus-free, episomal reprogramming protocol as described by Yu and colleagues.[1] iPSC-Heps were differentiated from iPSCs on multi-well plates coated with Matrigel® (Corning Life Sciences, Bedford, MA) using a modification of the Duncan protocol[2] developed by Dr. A.N. Mattis.[3] Definitive endoderm was induced using Activin A and doxycycline, followed by hepatic specification with FGF2, BMP4 and HGF, and maturation in hepatocyte culture medium (HCM™, Lonza) supplemented with oncostatin M. All experiments were performed at day 21 of differentiation. For Seahorse experiments, iPSCs were differentiated in Matrigel®-coated culture dishes until day 10, then detached with Accutase (Thermo Fisher Scientific, Waltham, MA) and replated at the same density in Seahorse XF24 V7 PS Cell Culture Microplates (Agilent, Santa Clara CA). Subject recruitment for iPSC reprogramming, as well as the propagation and differentiation of iPSCs for in vitro disease modeling, was approved by the Institutional Review Board and the Human Gamete, Embryo and Stem Cell Research Committee at the University of California San Francisco.

iPSC genotyping and calculation of a polygenic risk score. iPSCs were genotyped for 17 SNPs known to be associated with MASLD.[4] Genotypes for some SNPs were assessed by QPCR using commercial TaqMan™ assays (Applied Biosystems™, Waltham, MA) according to instructions recommended by the manufacturer; genotypes for other SNPs were determined by Sanger sequencing. The specific TaqMan™ assays or primers used for sequencing are shown in **Table S2;** subject genotypes are illustrated in **Table S3**. A weighted polygenic risk score was calculated for each iPSC line by multiplying the number of risk alleles for each variant by the effect size (β) and totaling the values for all 17 variants.

Neutral lipid stain. iPSC-Heps were fixed with 4% paraformaldehyde for 20 min at room temperature, stained with 1 μg/mL BODIPY 493/503 and counterstained with 10 μg/mL Hoechst 33342 in PBS for 1 h at room temperature and then washed once with PBS. Cells were imaged using a BioTek Cytation 5 imager.

Cellular triglyceride measurement. Triglyceride levels in iPSC-Heps were measured using the Triglyceride Assay Kit from BioVision (Milpitas, CA) according to the manufacturer's instructions. Briefly, iPSC-Heps were homogenized in 5% NP-40 and heated twice to 100 °C for 3 min to ensure lipid solubilization. Samples were then centrifuged at 16,000 × g and supernatants mixed with lipase for 20 min to release glycerol, which was measured using a Synergy HT microplate reader (BioTek, Winooski, VT). Results were normalized to cellular protein content (Pierce BCA Protein Assay, Thermo Fisher Scientific, Waltham, MA).

RNA sequencing. RNA sequencing was performed on the iPSC-Heps in this study as part of a larger cohort.[5] Differential gene expression was analyzed using edgeR by disease group, with

batch effect addressed as a covariate in a generalized linear model.[6] The RNA sequencing data set used in this study are deposited in GEO: GSE306392.

Studies of mitochondrial mass. Mitochondrial mass was assessed in live cells by staining with MitoTracker Green FM (Invitrogen, Waltham, MA) and measuring citrate synthase activity in whole cell extracts (Citrate Synthase Activity Assay Kit, Millipore Sigma, Burlington, MA). Enzyme activity was normalized to cellular protein content.

Oxygen consumption measurements. Mitochondrial oxygen consumption rates (OCR) in the presence of electron transport chain inhibitors and uncouplers (oligomycin, 1.5 μM; carbonyl cyanide-p-trifluoromethoxyphenylhydrazone (FCCP), 2 μM; rotenone, 0.5 μM; antimycin A, 0.5 μM) were measured in adherent iPSC-Heps using a Seahorse XFe24 Analyzer (Agilent, Santa Clara, CA). Two assays were performed: the Mito Stress Test and the Seahorse XF Palmitate Oxidation Stress Test (Agilent) according to the manufacturer's instructions. For the Mito Stress Test, fully differentiated iPSC-Heps were pre-incubated in DMEM supplemented with either 10 mM glucose and 2 mM glutamine (*glucose condition*) or 10 mM galactose and 6 mM glutamine (*galactose condition*) for 3 h to induce a metabolic switch.[7] OCR was then assessed under the same substrate conditions plus 1 mM pyruvate. For the Palmitate Oxidation Stress Test, iPSC-Heps were pre-incubated overnight in a substrate-limited medium containing 0.5 mM glucose, 0.5 mM L-carnitine and 1 mM GlutaMAX to promote fatty acid oxidation (FAO). Endogenous FAO was then assessed in the Seahorse Analyzer by comparing OCR in the presence and absence of etomoxir, an inhibitor of carnitine palmitoyltransferase-1. Exogenous FAO was assessed in parallel wells of iPSC-Heps by treating the cells with or without palmitate immediately prior to Seahorse analysis. Endogenous and exogenous FAO were calculated as the difference in OCR between cells treated with or without etomoxir and with or without palmitate, respectively. OCR

values were normalized to the number of cells in each well, assessed by counting stained nuclei (Hoechst 33342, Sigma-Aldrich) with CellProfiler image analysis software.

Assessment of mitochondrial ROS generation. Mitochondrial ROS following 2 h of BSA (control) or palmitate (350 μM or 700 μM) treatment was assessed using MitoSOX Red (3 μM), a fluorescent probe for mitochondrial superoxide. Fluorescence was measured using well-scanning spectrofluorimetry on a Synergy HT microplate reader at excitation/emission wavelengths of 520/590 nm. Data were expressed as percent of the respective BSA control.

Cellular energy status. ADP/ATP ratios in iPSC-Heps were measured using the ADP/ATP Ratio Assay Kit (Sigma-Aldrich) according to the manufacturer's instructions. Briefly, iPSC-Heps treated for 2 h with BSA or palmitate (700 μM) were lysed and luminescence measured before and after enzymatic conversion of ADP to ATP. The ADP/ATP ratio was then calculated from these measurements.

Statistical analyses. In all assays, outcome measures were assessed using iPSC-Heps from 6-10 MASLD and 6-10 control subjects. The number of subjects analyzed in each assay is indicated in the figure legends. Statistical comparisons between MASLD and control groups were made using the Student's t-test; linear regression was assessed using the F-test. In some cases, 2-way ANOVA was used with Šídák's post hoc test for multiple comparisons. P values < .05 were considered significant.

Author review and approval. All authors had access to the primary study data, and all authors reviewed and approved the final version of the manuscript.

Table S1. Subject Demographics

Sample ID	CIRM ID	Age	Sex	Ethnicity	BMI	DM2	St (0-3)	Bal (0-2)	Inf (0-3)	MAS (0-8)	Fib (0-4)	Pathologist Diagnosis
**Control 1**	CW10001	23	F	SA	19.2	N	--	--	--	--	--	--
**Control 2**	CW10002	23	F	EA	22.3	N	--	--	--	--	--	--
**Control 3**	CW10190	31	F	HW	25.4	N	--	--	--	--	--	--
**Control 4**	CW10192	32	F	HW	26.3	N	--	--	--	--	--	--
**Control 5**	CW10206	32	M	SA	22.4	N	--	--	--	--	--	--
**Control 6**	CW10173	37	F	AA	24.4	N	--	--	--	--	--	--
**Control 7**	CW10030	38	M	Mixed	21.2	N	--	--	--	--	--	--
**Control 8**	CW10149	38	M	NHPI	26.6	N	--	--	--	--	--	--
**Control 9**	CW10178	42	M	HW	27.5	N	--	--	--	--	--	--
**Control 10**	CW10037	47	M	NHW	25.0	N	--	--	--	--	--	--
**MASLD 1**	CW10022	30	F	NHW	32.7	N	3	0	0	3	0	MASL
**MASLD 2**	CW10182	38	M	SA	22.9	N	1	1	1	3	1	MASH
**MASLD 3**	CW10194	45	F	HW	37.9	N	2	2	1	5	3	MASH
**MASLD 4**	CW10172	47	F	HW	37.2	Y	1	2	1	4	3	MASH
**MASLD 5**	CW10166	48	M	HW	30.0	N	2	1	1	4	3	MASH
**MASLD 6**	CW10131	49	M	HW	22.1	Y	2	1	1	4	3	MASH
**MASLD 7**	CW10201	52	M	HW	29.9	Y	2	2	1	5	4	MASH
**MASLD 8**	CW10165	54	F	HW	43.7	Y	1	2	1	4	3	MASH
**MASLD 9**	CW10077	57	F	HW	58.1	Y	3	2	3	8	4	MASH
**MASLD 10**	CW10208	71	F	HW	36.0	pre	1	2	1	4	4	MASH

SA, South Asian; EA, East Asian; NHW, non-Hispanic White; NHPI, Native Hawaiian or other Pacific Islander; HW, Hispanic White; Bal, ballooning; DM2, type 2 diabetes; Fib, fibrosis; Inf, inflammation; MAS, MASLD Activity Score; St, steatosis; MASL, metabolic dysfunction-associated steatotic liver; MASH, metabolic dysfunction-associated steatohepatitis.

Table S2. TaqMan™ SNP genotyping assay IDs and Sanger sequencing primers used for SNP genotyping

TaqMan™ genotyping assays		
**Gene**	**rsID**	***Assay ID***
ADH1B	rs1229984	C___2688467_20
APOE	rs429358	C___3084793_20
COBLL1	rs79953491	C__99843946_10
FTO	rs17817449	C__34511515_10
GCKR	rs1260326 [a]	C___2862880_1_
GPAM	rs4918722	C__27410873_10
MARC1	rs2642438	C___1235772_10
MBOAT7 [a]	rs641738	C___8716820_10
MTTP	rs7661964	C_189405635_10
PNPLA2	rs140201358	C_161137417_10
PTPRD	rs10756038	C__31297394_10
TM6SF2	rs58542926	C__89463510_10
TOR1B	rs7029757	C__25765514_10
**Sanger sequencing primers**		
**Gene**	**rsID**	***Primer***
INSR (forward)	rs112630404	TGCACACTGAACTTCTTGCC
INSR (reverse)		TGCTCAAACAACAGGCAAGG
PNPLA3 (forward)	rs738409 [b]	GTTGAAGTTCACTGACAGGG
PNPLA3 (reverse)		CCAGCCAGTTTACCTTACAG
SREBF1 (forward)	rs4561528	TCTTGGTGGGCTCAAAGGAT
SREBF1 (reverse)		AGATGGATGCTGGACAGGTC

[a]This rsID is different from the rsID used by Chen et al[4] (rs626283), but the two are in linkage disequilibrium and are considered interchangeable.

[b]This rsID is different from the rsID used by Chen et al[4] (rs738408), but the two are in linkage disequilibrium and are considered interchangeable.

Table S3. Genotypes for MASLD-associated variants

Gene		ADH1B	APOE	COBLL1	FTO	GCKR	GPAM	INSR	MARC1	MBOAT7	MTTP	PNPLA2	PNPLA3	PTPRD	SREBF1	TM6SF2	TOR1B	TRIB1
**rsID [a]**		rs1229984	rs429358	rs79953491	rs17817449	rs1260326	rs4918722	rs112630404	rs2642438	rs641738	rs7661964	rs140201358	rs738409	rs10756038	rs4561528	rs58542926	rs7029757	rs112875651
**Effect allele**		C	T	A	G	T	C	A	G	T	A	G	G	G	T	T	G	G
**β [b]**		0.13	0.04	0.04	0.04	0.07	0.05	0.06	0.04	0.03	0.05	0.13	0.24	0.05	0.03	0.17	0.06	0.03
**GEO #**	**CIRM ID**																	
**Control 1**	CW1001	CC	TT	AA	TT	CC	CT	TT	AG	TT	AA	CC	GG	GA	TC	CC	GG	GG
**Control 2**	CW1002	TT	CT	AA	GT	CT	TT	TT	GG	CC	AA	CC	GG	AA	TT	CC	GG	GG
**Control 3**	CW10190	CC	TT	AA	TT	CT	TT	TT	AG	CC	AA	CC	CG	GA	TC	CC	GG	GA
**Control 4**	CW10192	CC	TT	AA	GT	CT	CT	TT	GG	TT	AA	CC	GG	GG	TT	CC	GG	GG
**Control 5**	CW10206	CC	TT	AA	GT	CT	TT	TT	GG	CC	AT	CC	GG	GA	TT	CC	GA	GA
**Control 6**	CW10173	CC	TT	AG	TT	CT	TT	TT	GG	CC	AA	CC	CC	GG	CC	CC	GG	AA
**Control 7**	CW10030	CC	CT	AA	GG	CT	CT	TT	GG	CC	AT	CC	CC	GG	TT	CC	GG	GG
**Control 8**	CW10149	CT	TT	AA	TT	CT	TT	TT	AG	TT	AT	CC	CC	GA	TC	CC	GG	GA
**Control 9**	CW10178	CC	CT	AA	GT	CT	CT	TT	AG	CC	TT	CC	CG	GG	TC	CC	GA	AA
**Control 10**	CW10037	CC	TT	AA	GT	CT	CT	TT	GG	TT	AA	CC	GG	GG	CC	CT	GG	GG
**MASLD 1**	CW10022	CC	CT	AA	TT	CT	TT	TT	AA	CC	AT	CC	GG	GA	CC	CC	GG	AA
**MASLD 2**	CW10182	CC	TT	AA	GT	CT	TT	TT	GG	TT	TT	CC	CG	AA	TT	CC	GG	GA
**MASLD 3**	CW10194	CC	TT	AA	TT	CT	CT	TT	GG	CC	AT	CC	GG	GA	TC	CT	GG	GG
**MASLD 4**	CW10172	CC	TT	AA	TT	CT	CT	AT	GG	TT	AT	CC	GG	GG	CC	CC	GG	AA
**MASLD 5**	CW10166	CC	TT	AA	TT	CT	CT	AT	GG	CC	AA	CC	GG	GG	TC	CC	GG	GA
**MASLD 6**	CW10131	CC	TT	AA	GG	CT	TT	TT	GG	CC	AA	CC	CG	AA	TC	CC	GG	GG
**MASLD 7**	CW10201	CC	TT	AA	GT	CT	TT	TT	GG	TT	AA	CC	GG	GG	TT	CT	GG	GA
**MASLD 8**	CW10165	CC	TT	AA	TT	CC	TT	TT	AG	CC	AA	CC	GG	GG	CC	CT	GG	GG
**MASLD 9**	CW1077	CC	TT	AA	TT	TT	CT	AT	GG	TT	AA	CC	GG	GG	TC	CC	GG	GG
**MASLD 10**	CW10208	CT	CT	AA	GT	CT	CT	AT	AG	TT	AA	CC	GG	AA	TT	CT	GG	GG

[a] rsIDs for MBOAT7 and PNPLA3 are different from those reported by Chen et al;[4] see Table S2.
[a] Effect sizes are those reported by Chen et al[4] for all ancestries.

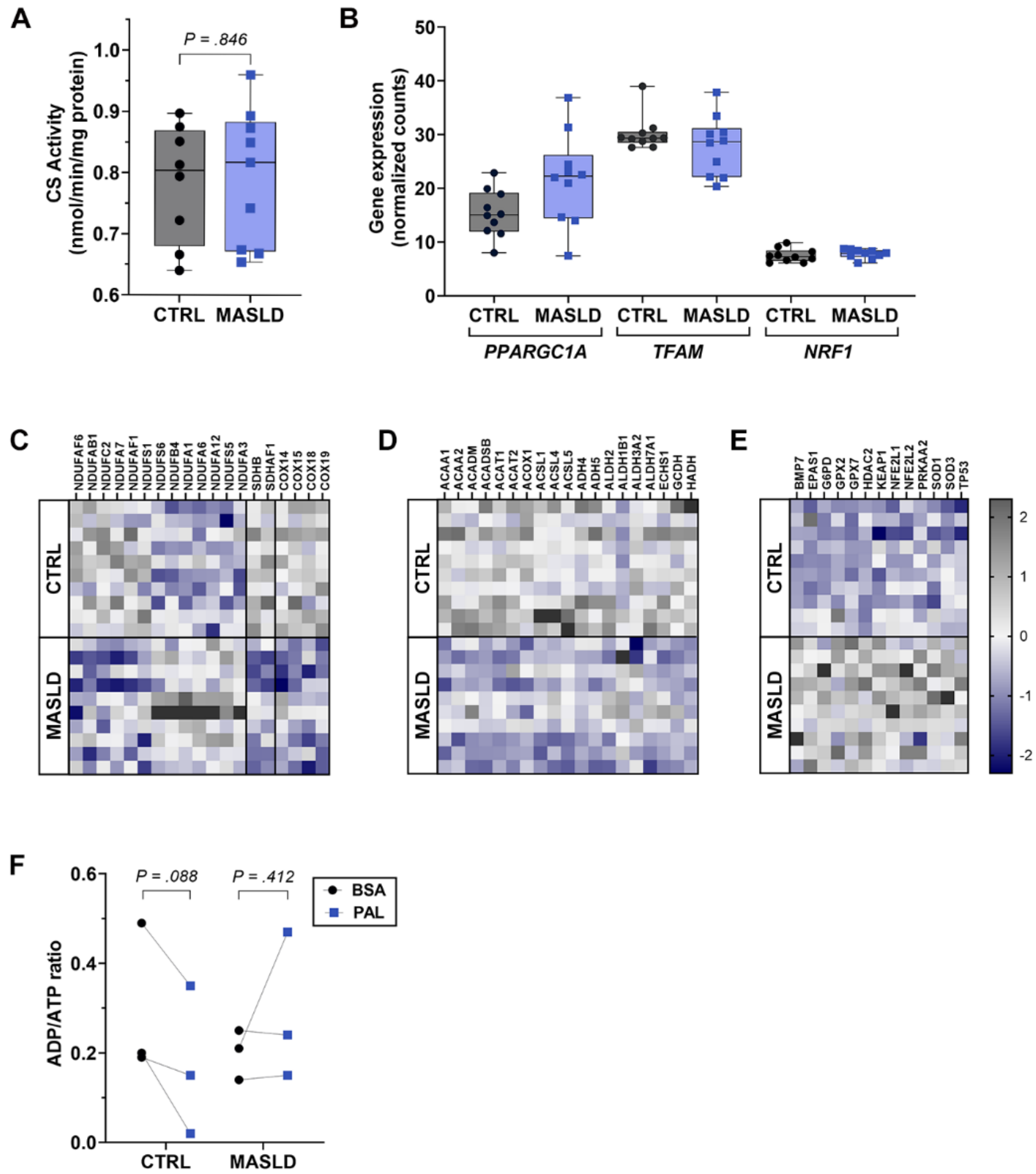

Figure S1. Assays of mitochondrial integrity, displays of differential gene expression, and measurement of ATP synthesis in MASLD vs. control iPSC-Heps.

(A) Citrate synthase (CS) activity in MASLD and CTRL iPSC-Heps. **(B)** Expression of 3 genes involved in mitochondrial biogenesis, measured in MASLD and CTRL iPSC-Heps at day 21 of differentiation. *PPARGC1A*, peroxisome proliferator-activated receptor gamma coactivator 1-alpha; *TFAM*, transcription factor A, mitochondrial; *NRF1*, nuclear respiratory factor 1. **(C-E)** Heatmaps illustrating genes involved in **(C)** oxidative phosphorylation, **(D)** fatty acid oxidation and **(E)** responses to oxidative stress that are differentially expressed ($P < .05$) in MASLD vs. CTRL iPSC-Heps. **(F)** ADP/ATP ratio in MASLD and control iPSC-Heps following treatment with BSA or 700 µM palmitate (PAL) as indicator of fatty acid-induced ATP synthesis. *P* values represent the results of paired t-tests.

REFERENCES

1. Yu J, Chau KF, Vodyanik MA, Jiang J, Jiang Y. Efficient feeder-free episomal reprogramming with small molecules. PLoS One 2011;6:e17557.
2. Si-Tayeb K, Noto FK, Nagaoka M, Li J, Battle MA, Duris C, North PE, Dalton S, Duncan SA. Highly efficient generation of human hepatocyte-like cells from induced pluripotent stem cells. Hepatology 2010;51:297–305.
3. Peaslee C, Esteva-Font C, Su T, Munoz-Howell A, Duwaerts CC, Liu Z, Rao S, Liu K, Medina M, Sneddon JB, Maher JJ, Mattis AN. Doxycycline Significantly Enhances Induction of Induced Pluripotent Stem Cells to Endoderm by Enhancing Survival Through Protein Kinase B Phosphorylation. Hepatology 2021;74:2102–2117.
4. Chen Y, Du X, Kuppa A, Feitosa MF, Bielak LF, O'Connell JR, Musani SK, Guo X, Kahali B, Chen VL, Smith AV, Ryan KA, Eirksdottir G, Allison MA, Bowden DW, Budoff MJ, Carr JJ, Chen YI, Taylor KD, Oliveri A, Correa A, Crudup BF, Kardia SLR, Mosley TH, Jr., Norris JM, Terry JG, Rotter JI, Wagenknecht LE, Halligan BD, Young KA, Hokanson JE, Washko GR, Gudnason V, Province MA, Peyser PA, Palmer ND, Speliotes EK. Genome-wide association meta-analysis identifies 17 loci associated with nonalcoholic fatty liver disease. Nat Genet 2023;55:1640–1650.
5. Duwaerts CC, Le Guillou D, Her CL, Phillips NJ, Willenbring H, Mattis AN, Maher JJ. Induced Pluripotent Stem Cell-derived Hepatocytes From Patients With Nonalcoholic Fatty Liver Disease Display a Disease-specific Gene Expression Profile. Gastroenterology 2021;160:2591–2594 e6.
6. Robinson MD, McCarthy DJ, Smyth GK. edgeR: a Bioconductor package for differential expression analysis of digital gene expression data. Bioinformatics 2010;26:139–40.
7. Kamalian L, Douglas O, Jolly CE, Snoeys J, Simic D, Monshouwer M, Williams DP, Park BK, Chadwick AE. Acute Metabolic Switch Assay Using Glucose/Galactose Medium in HepaRG Cells to Detect Mitochondrial Toxicity. Curr Protoc Toxicol 2019;80:e76.

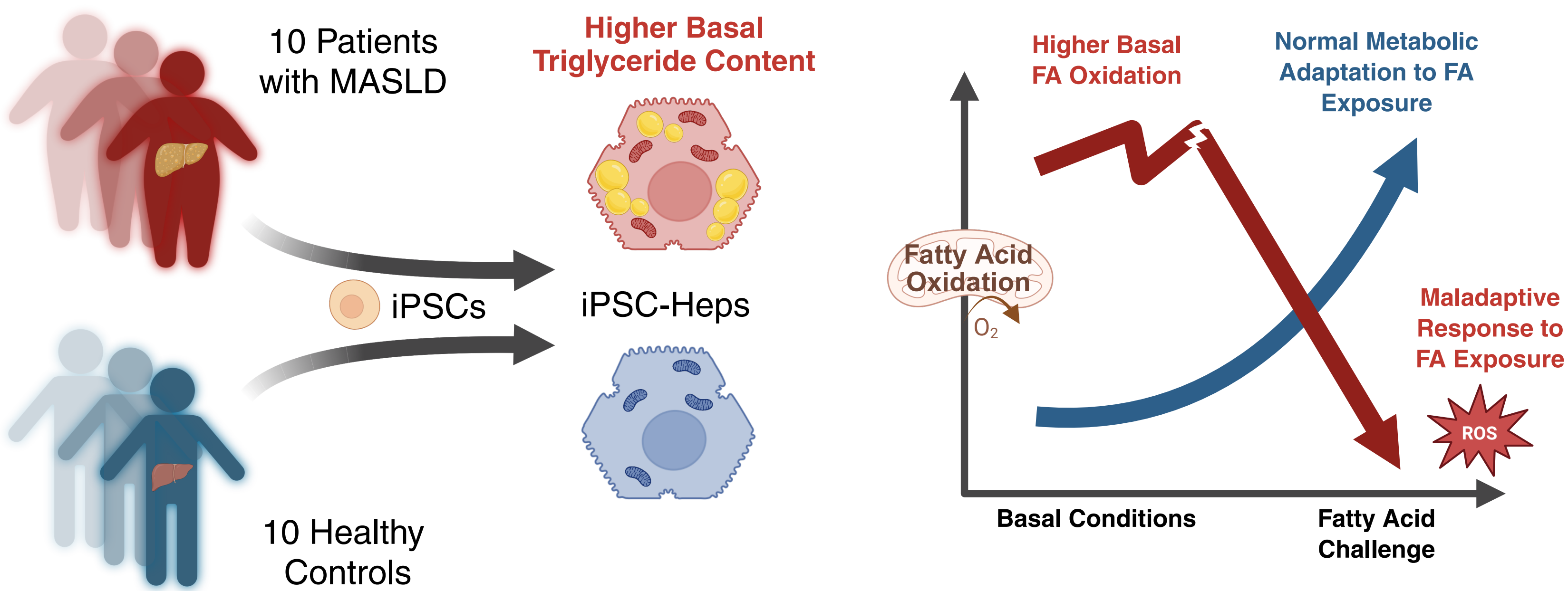

Created in BioRender. Maher, J. (2026) https://BioRender.com/dmf1zqg

cmgh CELLULAR AND MOLECULAR GASTROENTEROLOGY AND HEPATOLOGY
