# Supplementary material for: Induced Pluripotent Stem Cell–Derived Hepatocytes From Patients With Metabolic Dysfunction–Associated Steatotic Liver Disease Exhibit Early Mitochondrial Dysfunction
Source: Cell Mol Gastroenterol Hepatol. 2026 Aug 13;21(1):101853. doi: 10.1016/j.jcmgh.2026.101853 (PMC13677320; doi:10.1016/j.jcmgh.2026.101853)
Supplement: Supplementary file 2 [file mmc2.pdf]

cmgh
CELLULAR AND MOLECULAR GASTROENTEROLOGY AND HEPATOLOGY

RESEARCH LETTERS

Induced Pluripotent Stem Cell–Derived Hepatocytes From Patients With Metabolic Dysfunction–Associated Steatotic Liver Disease Exhibit Early Mitochondrial Dysfunction

Mitochondria are central to cellular energy production and homeostasis. In metabolic dysfunction–associated steatotic liver disease (MASLD), hepatocellular lipid accumulation triggers mitochondrial stress by increasing the demand for fatty acid oxidation (FAO).[1] In early MASLD, mitochondria adapt to lipid accumulation by raising their oxidative capacity and activating antioxidant defenses[2–5]; however, as metabolic dysfunction–associated steatotic liver transitions toward metabolic dysfunction–associated steatohepatitis (MASH), this adaptation deteriorates,[6] resulting in the accumulation of harmful reactive oxygen species (ROS). Excess mitochondrial ROS promote lipid peroxidation and activate apoptotic and inflammatory signaling pathways.[7] Moreover, ROS can impair mitochondrial biogenesis, resulting in a reduction of functioning mitochondrial mass. These observations underscore the importance of mitochondrial ROS as drivers of MASLD and MASH.

Our group uses induced pluripotent stem cell–derived hepatocytes (iPSC-Heps) to model human MASLD. We recently reported that iPSC-Heps from patients with biopsy-proven MASLD spontaneously develop steatosis and display a transcriptomic signature distinct from healthy subjects.[8] In this study, we evaluated mitochondrial function in patient-derived MASLD iPSC-Heps to determine whether they recapitulate the mitochondrial disturbances observed in MASLD livers in vivo.

We selected induced pluripotent stem cell (iPSC) lines from 10 patients with MASLD and 10 healthy control subjects whose transcriptomic profiles after differentiation to iPSC-Heps clearly placed them into distinct groups.[8] The demographic features of the iPSC donors are shown in Supplementary Table 1. Following differentiation of iPSCs to iPSC-Heps, MASLD iPSCs displayed spontaneous triglyceride (TG) accumulation, whereas control iPSC-Heps did not (Figure 1*A* and *B*). There was no difference in mitochondrial mass (Figure 1*C*) between MASLD and control iPSC-Heps, nor was there any difference between the 2 groups in the activity of citrate synthase or the

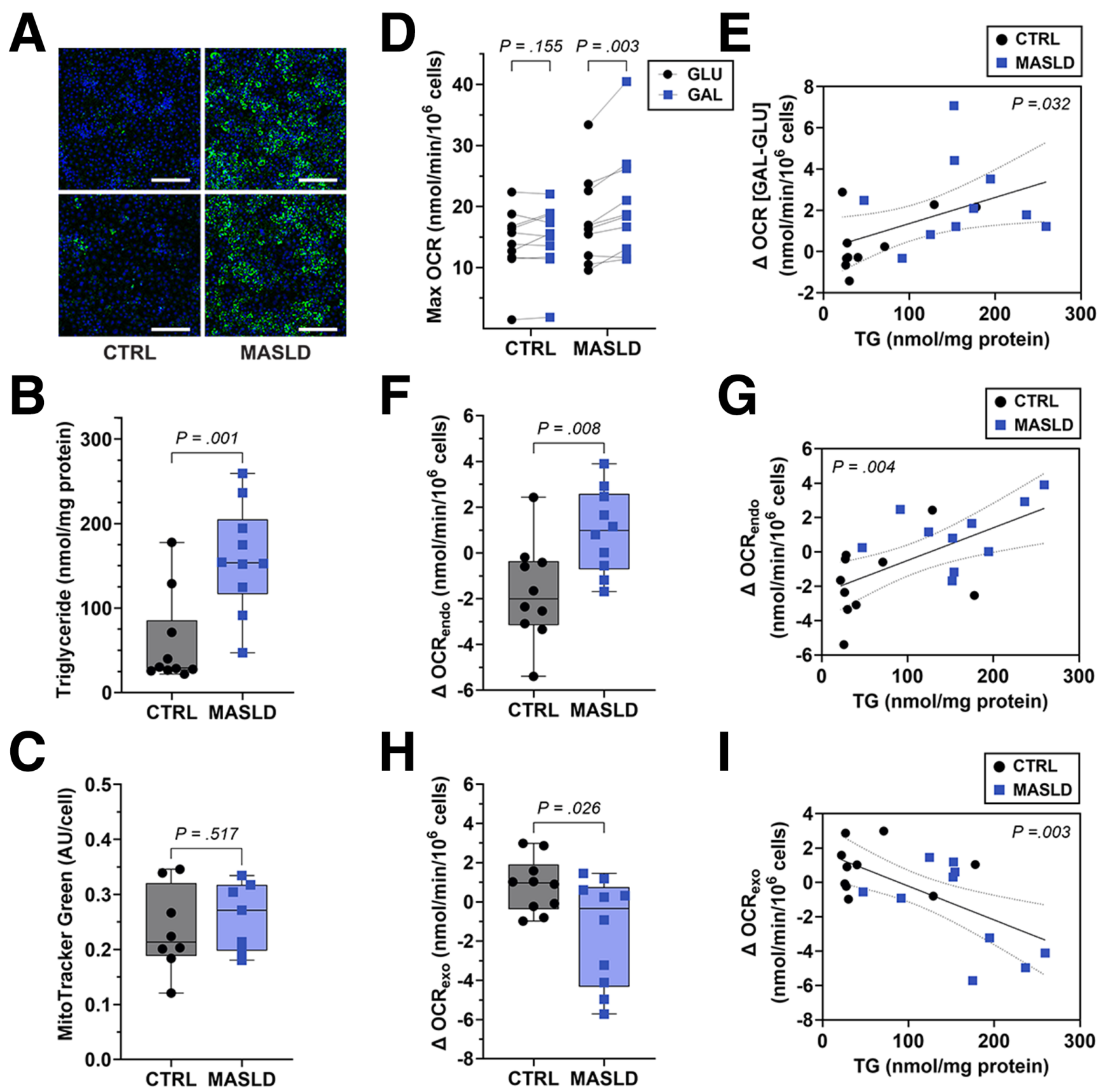

Figure 1. Lipid accumulation and mitochondrial function in metabolic dysfunction–associated steatotic liver disease (MASLD) vs control (CTRL) induced pluripotent stem cell–derived hepatocytes (iPSC-Heps). (*A*) Lipid droplets in 2 MASLD and 2 CTRL iPSC-Heps stained with BODIPY 493/503. Scale bar, 200 μm. (*B*) Cellular triglyceride (TG) content. (*C*) Mitochondrial mass based on MitoTracker Green staining (N = 8 CTRL and 7 MASLD). (*D* and *E*) Maximal oxygen consumption rate (OCR) in glucose (GLU) or galactose (GAL) and correlation between Δ OCR [GAL-GLU] and TG content. (*F* and *G*) Maximal endogenous fatty acid oxidation (FAO)-linked OCR and correlation with TG content. (*H* and *I*) Maximal exogenous FAO-linked OCR and correlation with TG content. Unless otherwise indicated, N = 10 CTRL and N = 10 MASLD; each symbol represents 1 subject. *P* values in (*B*, *C*, *F* and *H*) represent unpaired and (*D*) paired Student *t* tests. *P* values in (*E*, *G* and *I*) represent F-tests; *dotted lines* represent 95% confidence intervals.

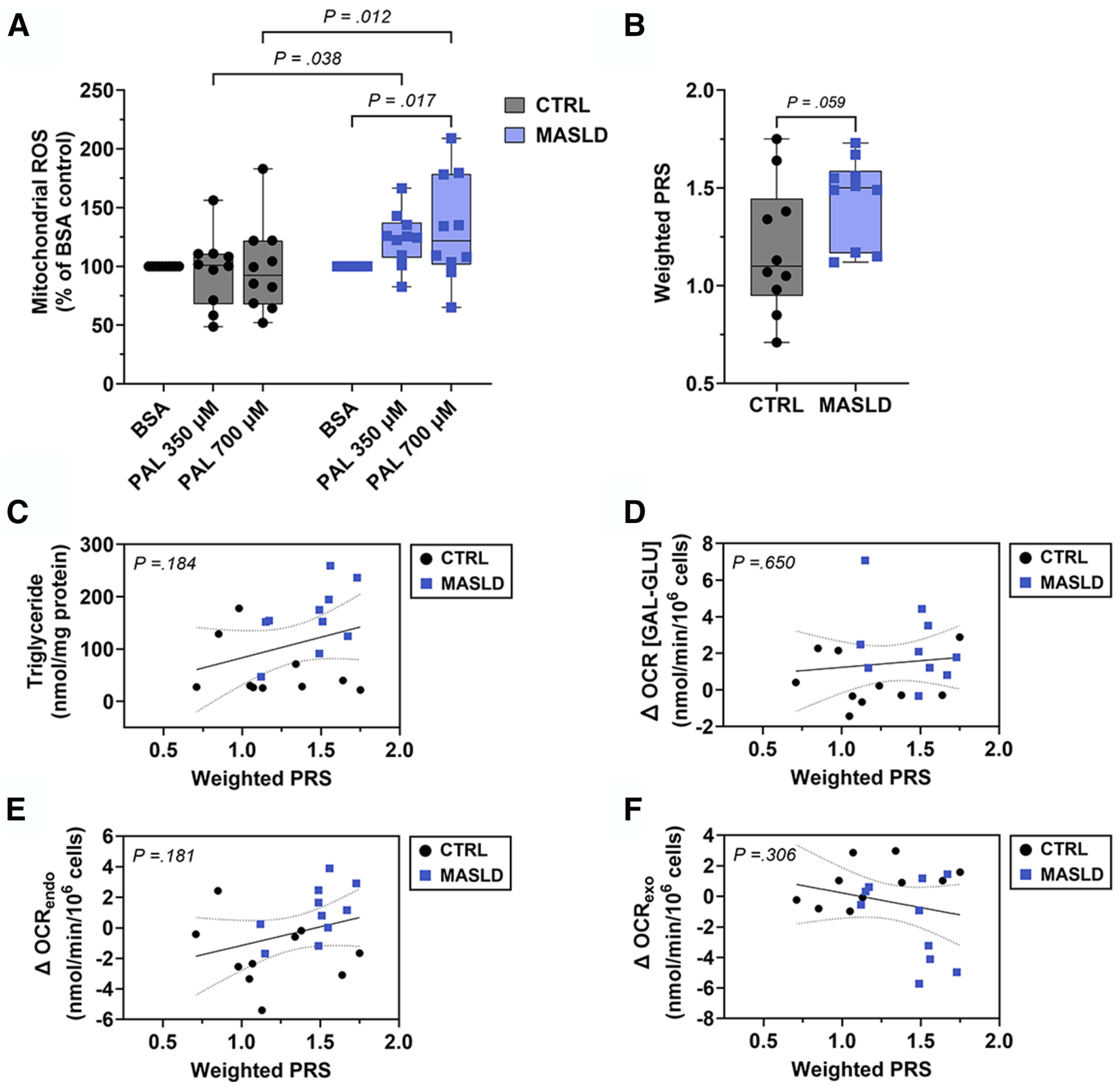

Figure 2. Mitochondrial reactive oxygen species (ROS) production in induced pluripotent stem cell–derived hepatocytes (iPSC-Heps); the impact of polygenic risk on the iPSC-Hep phenotype. (*A*) Mitochondrial ROS production in iPSC-Heps treated with bovine serum albumin (BSA) or palmitate (PAL). *P* values from 2-way analysis of variance with Šídák's post hoc testing. (*B*) Weighted polygenic risk score (PRS) in control (CTRL) and metabolic dysfunction–associated steatotic liver disease (MASLD) iPSCs. *P* value from unpaired Student *t* test. (*C–F*) Correlations between weighted PRS and triglyceride (TG) content (*C*), mitochondrial oxygen consumption rate (OCR) (*D*), and endogenous (*E*) and exogenous (*F*) fatty acid oxidation (FAO). *P* values from F-tests; *dotted* lines represent 95% confidence intervals. N = 10 CTRL and 10 MASLD; each symbol represents 1 subject. GAL, galactose; GLU, glucose.

expression of genes related to mitochondrial biogenesis (Supplementary Figure 1*A* and *B*). We evaluated mitochondrial oxygen consumption rates (OCRs) and found that MASLD, but not control, iPSC-Heps significantly increased their maximal OCR when cultured with galactose to force the use of oxidative metabolism for energy production (Figure 1*D*). The change in OCR between glucose and galactose conditions correlated positively with intracellular TG content (Figure 1*E*) and was associated with the reduced expression of genes in oxidative phosphorylation (OXPHOS) complexes II and IV (Supplementary Figure 1*C*).

To determine whether FAO is dysregulated in MASLD vs control iPSC-Heps, we measured FAO-linked OCR in the setting of endogenous and exogenous fatty acid. Endogenous FAO-linked OCR was higher in MASLD than control iPSC-Heps (Figure 1*F*) and correlated positively with iPSC-Hep TG content (Figure 1*G*). This was true despite the downregulation of several genes involved in fatty acid degradation (Supplementary Figure 1*D*). In contrast, when iPSC-Heps were challenged with exogenous palmitate, FAO-linked OCR was significantly lower in MASLD than control iPSC-Heps (Figure 1*H*) and correlated negatively with cellular TG content (Figure 1*I*). The latter result suggests that MASLD iPSC-Heps, which already contain excess lipid, are unable to accommodate an exogenous lipid challenge, signaling mitochondrial dysfunction. MASLD iPSC-Heps also produced significantly more ROS and less adenosine triphosphate than control iPSC-Heps when challenged with palmitate (Figure 2*A*; Supplementary Figure 1*F*), despite an increase in the expression of several genes involved in antioxidant defense (Supplementary Figure 1*E*).

When viewed in aggregate, our functional studies indicate that iPSC-Heps from patients with MASLD display disturbances in mitochondrial respiration resembling those in the intact human liver during the early stages of MASLD.[3] Specifically, MASLD iPSC-Heps have a low threshold for utilizing endogenous fatty acids for energy production but have difficulty coping with an exogenous fatty acid load, resulting in ROS production and reduced adenosine triphosphate generation. This mimics findings in rodents[9] and humans[2,3,5] in which there is an early increase in mitochondrial respiration during the development of MASLD that is then lost in MASH.

Genetic risk has been implicated in the occurrence of hepatic mitochondrial

alterations in humans with MASLD.[10] We evaluated genetic risk in our cohort by calculating a weighted polygenic risk score (PRS) for each subject. Although there was a trend toward higher PRS in the MASLD group, it did not reach statistical significance (Figure 2*B*). When we assessed the metabolic phenotypes of iPSC-Heps as a function of PRS, we found no correlation (Figure 2*C–F*). These findings suggest that genetic background, at least with respect to the known single nucleotide polymorphisms tested, is not the sole determinant of MASLD-related metabolic dysfunction in iPSC-Heps.

A key strength of our study is that we reproduced features of human MASLD in patient-derived iPSC-Heps that heretofore could only be studied in liver tissue. Moreover, we demonstrated that mitochondrial alterations are detectable in iPSC-Heps from several unrelated donors with MASLD, underscoring the utility of population-based iPSC modeling to investigate disease pathogenesis. A limitation of the study is the small number of subjects in each group.

In summary, iPSC-Heps from patients with biopsy-proven MASLD exhibit mitochondrial alterations representative of early MASLD in vivo. Several of these alterations arise even without an exogenous metabolic challenge, indicating that MASLD iPSC-Heps are intrinsically susceptible to metabolic stress. Importantly, mitochondrial dysfunction in MASLD iPSC-Heps does not correlate directly with genetic risk. This is seemingly at odds with human data in vivo[10] and warrants further investigation.

DOUNIA LE GUILLOU
KEVIN SIAO
CHRIS L. HER
CAROLINE C. DUWAERTS
JACQUELYN J. MAHER
Department of Medicine
University of California San Francisco
San Francisco, California, *and*
The Liver Center
University of California San Francisco
San Francisco, California

Supplementary Material

Note: To access the supplementary material accompanying this article, visit the full text version at https://doi.org/10.1016/j.jcmgh.2026.101853.

References

1. Fromenty B, et al. J Hepatol 2023;78:415–429.
2. Sunny NE, et al. Cell Metab 2011;14:804–810.
3. Koliaki C, et al. Cell Metab 2015;21:739–746.
4. Moore MP, et al. Hepatology 2022;76:1452–1465.
5. Greatorex S, et al. J Clin Invest 2023;134.
6. Hyotylainen T, et al. Nat Commun 2016;7:8994.
7. Satapati S, et al. J Clin Invest 2015;125:4447–4462.
8. Duwaerts CC, et al. Gastroenterology 2021;160:2591–2594.e6.
9. Satapati S, et al. J Lipid Res 2012;53:1080–1092.
10. Luukkonen PK, et al. Cell Metab 2023;35:1887–1896.e5.

Most current article

© 2026 The Authors. Published by Elsevier Inc. on behalf of American Gastroenterological Association Institute. This is an open access article under the CC BY license (http://creativecommons.org/licenses/by/4.0/).

2352-345X
https://doi.org/10.1016/j.jcmgh.2026.101853

Correspondence
Address correspondence to: Dounia Le Guillou, PhD, 513 Parnassus Avenue, Box 0538, Health Sciences East 1426, San Francisco, California 94143. e-mail: dounia.leguillou@ucsf.edu; or Jacquelyn J. Maher, MD, 513 Parnassus Avenue, Box 0538, Health Sciences East 1426, San Francisco, California 94143. e-mail: jacquelyn.maher@ucsf.edu.

Conflicts of interest
The authors disclose no conflicts.

Funding
This study was supported by the California Institute for Regenerative Medicine (IT1-06563), the National Institutes of Health (NIH; R21DK118380, RC2DK136052, and P30DK026743).

Data Availability
Data and methods underlying this manuscript will be made available to other researchers upon request. The data discussed in this publication have been deposited in NCBI's Gene Expression Omnibus and are accessible through GEO Series accession number GSE306392.

Cellular and Molecular Gastroenterology and Hepatology, Volume *21*

Supplemental information

Induced Pluripotent Stem Cell–Derived Hepatocytes From Patients With Metabolic Dysfunction–Associated Steatotic Liver Disease Exhibit Early Mitochondrial Dysfunction

Dounia Le Guillou, Kevin Siao, Chris L. Her, Caroline C. Duwaerts, and Jacquelyn J. Maher

Supplemental Information for

Induced pluripotent stem cell-derived hepatocytes from patients with MASLD exhibit early mitochondrial dysfunction

Dounia Le Guillou,[1,2] Kevin Siao,[1,2] Chris L. Her,[1,2] Caroline C. Duwaerts[1,2] and Jacquelyn J. Maher[1,2]

[1]Department of Medicine and [2]Liver Center, University of California, San Francisco

METHODS

Generation of iPSCs and iPSC-Heps. Peripheral blood mononuclear cells were used to generate iPSCs from all subjects. iPSCs were reprogrammed by Cellular Dynamics International, Inc. (Novato, CA) using a virus-free, episomal reprogramming protocol as described by Yu and colleagues.[1] iPSC-Heps were differentiated from iPSCs on multi-well plates coated with Matrigel® (Corning Life Sciences, Bedford, MA) using a modification of the Duncan protocol[2] developed by Dr. A.N. Mattis.[3] Definitive endoderm was induced using Activin A and doxycycline, followed by hepatic specification with FGF2, BMP4 and HGF, and maturation in hepatocyte culture medium (HCM™, Lonza) supplemented with oncostatin M. All experiments were performed at day 21 of differentiation. For Seahorse experiments, iPSCs were differentiated in Matrigel®-coated culture dishes until day 10, then detached with Accutase (Thermo Fisher Scientific, Waltham, MA) and replated at the same density in Seahorse XF24 V7 PS Cell Culture Microplates (Agilent, Santa Clara CA). Subject recruitment for iPSC reprogramming, as well as the propagation and differentiation of iPSCs for in vitro disease modeling, was approved by the Institutional Review Board and the Human Gamete, Embryo and Stem Cell Research Committee at the University of California San Francisco.

iPSC genotyping and calculation of a polygenic risk score. iPSCs were genotyped for 17 SNPs known to be associated with MASLD.[4] Genotypes for some SNPs were assessed by QPCR using commercial TaqMan™ assays (Applied Biosystems™, Waltham, MA) according to instructions recommended by the manufacturer; genotypes for other SNPs were determined by Sanger sequencing. The specific TaqMan™ assays or primers used for sequencing are shown in **Table S2;** subject genotypes are illustrated in **Table S3**. A weighted polygenic risk score was calculated for each iPSC line by multiplying the number of risk alleles for each variant by the effect size (β) and totaling the values for all 17 variants.

Neutral lipid stain. iPSC-Heps were fixed with 4% paraformaldehyde for 20 min at room temperature, stained with 1 µg/mL BODIPY 493/503 and counterstained with 10 µg/mL Hoechst 33342 in PBS for 1 h at room temperature and then washed once with PBS. Cells were imaged using a BioTek Cytation 5 imager.

Cellular triglyceride measurement. Triglyceride levels in iPSC-Heps were measured using the Triglyceride Assay Kit from BioVision (Milpitas, CA) according to the manufacturer's instructions. Briefly, iPSC-Heps were homogenized in 5% NP-40 and heated twice to 100 °C for 3 min to ensure lipid solubilization. Samples were then centrifuged at 16,000 × g and supernatants mixed with lipase for 20 min to release glycerol, which was measured using a Synergy HT microplate reader (BioTek, Winooski, VT). Results were normalized to cellular protein content (Pierce BCA Protein Assay, Thermo Fisher Scientific, Waltham, MA).

RNA sequencing. RNA sequencing was performed on the iPSC-Heps in this study as part of a larger cohort.[5] Differential gene expression was analyzed using edgeR by disease group, with

batch effect addressed as a covariate in a generalized linear model.[6] The RNA sequencing data set used in this study are deposited in GEO: GSE306392.

Studies of mitochondrial mass. Mitochondrial mass was assessed in live cells by staining with MitoTracker Green FM (Invitrogen, Waltham, MA) and measuring citrate synthase activity in whole cell extracts (Citrate Synthase Activity Assay Kit, Millipore Sigma, Burlington, MA). Enzyme activity was normalized to cellular protein content.

Oxygen consumption measurements. Mitochondrial oxygen consumption rates (OCR) in the presence of electron transport chain inhibitors and uncouplers (oligomycin, 1.5 μM; carbonyl cyanide-p-trifluoromethoxyphenylhydrazone (FCCP), 2 μM; rotenone, 0.5 μM; antimycin A, 0.5 μM) were measured in adherent iPSC-Heps using a Seahorse XFe24 Analyzer (Agilent, Santa Clara, CA). Two assays were performed: the Mito Stress Test and the Seahorse XF Palmitate Oxidation Stress Test (Agilent) according to the manufacturer's instructions. For the Mito Stress Test, fully differentiated iPSC-Heps were pre-incubated in DMEM supplemented with either 10 mM glucose and 2 mM glutamine (*glucose condition*) or 10 mM galactose and 6 mM glutamine (*galactose condition*) for 3 h to induce a metabolic switch.[7] OCR was then assessed under the same substrate conditions plus 1 mM pyruvate. For the Palmitate Oxidation Stress Test, iPSC-Heps were pre-incubated overnight in a substrate-limited medium containing 0.5 mM glucose, 0.5 mM L-carnitine and 1 mM GlutaMAX to promote fatty acid oxidation (FAO). Endogenous FAO was then assessed in the Seahorse Analyzer by comparing OCR in the presence and absence of etomoxir, an inhibitor of carnitine palmitoyltransferase-1. Exogenous FAO was assessed in parallel wells of iPSC-Heps by treating the cells with or without palmitate immediately prior to Seahorse analysis. Endogenous and exogenous FAO were calculated as the difference in OCR between cells treated with or without etomoxir and with or without palmitate, respectively. OCR

values were normalized to the number of cells in each well, assessed by counting stained nuclei (Hoechst 33342, Sigma-Aldrich) with CellProfiler image analysis software.

Assessment of mitochondrial ROS generation. Mitochondrial ROS following 2 h of BSA (control) or palmitate (350 μM or 700 μM) treatment was assessed using MitoSOX Red (3 μM), a fluorescent probe for mitochondrial superoxide. Fluorescence was measured using well-scanning spectrofluorimetry on a Synergy HT microplate reader at excitation/emission wavelengths of 520/590 nm. Data were expressed as percent of the respective BSA control.

Cellular energy status. ADP/ATP ratios in iPSC-Heps were measured using the ADP/ATP Ratio Assay Kit (Sigma-Aldrich) according to the manufacturer's instructions. Briefly, iPSC-Heps treated for 2 h with BSA or palmitate (700 μM) were lysed and luminescence measured before and after enzymatic conversion of ADP to ATP. The ADP/ATP ratio was then calculated from these measurements.

Statistical analyses. In all assays, outcome measures were assessed using iPSC-Heps from 6-10 MASLD and 6-10 control subjects. The number of subjects analyzed in each assay is indicated in the figure legends. Statistical comparisons between MASLD and control groups were made using the Student's t-test; linear regression was assessed using the F-test. In some cases, 2-way ANOVA was used with Šídák's post hoc test for multiple comparisons. *P* values < .05 were considered significant.

Author review and approval. All authors had access to the primary study data, and all authors reviewed and approved the final version of the manuscript.

Table S1. Subject Demographics

Sample ID	CIRM ID	Age	Sex	Ethnicity	BMI	DM2	St (0-3)	Bal (0-2)	Inf (0-3)	MAS (0-8)	Fib (0-4)	Pathologist Diagnosis
**Control 1**	CW10001	23	F	SA	19.2	N	--	--	--	--	--	--
**Control 2**	CW10002	23	F	EA	22.3	N	--	--	--	--	--	--
**Control 3**	CW10190	31	F	HW	25.4	N	--	--	--	--	--	--
**Control 4**	CW10192	32	F	HW	26.3	N	--	--	--	--	--	--
**Control 5**	CW10206	32	M	SA	22.4	N	--	--	--	--	--	--
**Control 6**	CW10173	37	F	AA	24.4	N	--	--	--	--	--	--
**Control 7**	CW10030	38	M	Mixed	21.2	N	--	--	--	--	--	--
**Control 8**	CW10149	38	M	NHPI	26.6	N	--	--	--	--	--	--
**Control 9**	CW10178	42	M	HW	27.5	N	--	--	--	--	--	--
**Control 10**	CW10037	47	M	NHW	25.0	N	--	--	--	--	--	--
**MASLD 1**	CW10022	30	F	NHW	32.7	N	3	0	0	3	0	MASL
**MASLD 2**	CW10182	38	M	SA	22.9	N	1	1	1	3	1	MASH
**MASLD 3**	CW10194	45	F	HW	37.9	N	2	2	1	5	3	MASH
**MASLD 4**	CW10172	47	F	HW	37.2	Y	1	2	1	4	3	MASH
**MASLD 5**	CW10166	48	M	HW	30.0	N	2	1	1	4	3	MASH
**MASLD 6**	CW10131	49	M	HW	22.1	Y	2	1	1	4	3	MASH
**MASLD 7**	CW10201	52	M	HW	29.9	Y	2	2	1	5	4	MASH
**MASLD 8**	CW10165	54	F	HW	43.7	Y	1	2	1	4	3	MASH
**MASLD 9**	CW10077	57	F	HW	58.1	Y	3	2	3	8	4	MASH
**MASLD 10**	CW10208	71	F	HW	36.0	pre	1	2	1	4	4	MASH

SA, South Asian; EA, East Asian; NHW, non-Hispanic White; NHPI, Native Hawaiian or other Pacific Islander; HW, Hispanic White; Bal, ballooning; DM2, type 2 diabetes; Fib, fibrosis; Inf, inflammation; MAS, MASLD Activity Score; St, steatosis; MASL, metabolic dysfunction-associated steatotic liver; MASH, metabolic dysfunction-associated steatohepatitis.

Table S2. TaqMan™ SNP genotyping assay IDs and Sanger sequencing primers used for SNP genotyping

TaqMan™ genotyping assays		
**Gene**	**rsID**	***Assay ID***
ADH1B	rs1229984	C___2688467_20
APOE	rs429358	C___3084793_20
COBLL1	rs79953491	C__99843946_10
FTO	rs17817449	C__34511515_10
GCKR	rs1260326 [a]	C___2862880_1_
GPAM	rs4918722	C__27410873_10
MARC1	rs2642438	C___1235772_10
MBOAT7 [a]	rs641738	C___8716820_10
MTTP	rs7661964	C_189405635_10
PNPLA2	rs140201358	C_161137417_10
PTPRD	rs10756038	C__31297394_10
TM6SF2	rs58542926	C__89463510_10
TOR1B	rs7029757	C__25765514_10
**Sanger sequencing primers**		
**Gene**	**rsID**	***Primer***
INSR (forward)	rs112630404	TGCACACTGAACTTCTTGCC
INSR (reverse)		TGCTCAAACAACAGGCAAGG
PNPLA3 (forward)	rs738409 [b]	GTTGAAGTTCACTGACAGGG
PNPLA3 (reverse)		CCAGCCAGTTTACCTTACAG
SREBF1 (forward)	rs4561528	TCTTGGTGGGCTCAAAGGAT
SREBF1 (reverse)		AGATGGATGCTGGACAGGTC

[a]This rsID is different from the rsID used by Chen et al[4] (rs626283), but the two are in linkage disequilibrium and are considered interchangeable.

[b]This rsID is different from the rsID used by Chen et al[4] (rs738408), but the two are in linkage disequilibrium and are considered interchangeable.

Table S3. Genotypes for MASLD-associated variants

Gene		ADH1B	APOE	COBLL1	FTO	GCKR	GPAM	INSR	MARC1	MBOAT7	MTTP	PNPLA2	PNPLA3	PTPRD	SREBF1	TM6SF2	TOR1B	TRIB1
rsID [a]		rs1229984	rs429358	rs79953491	rs17817449	rs1260326	rs4918722	rs112630404	rs2642438	rs641738	rs7661964	rs140201358	rs738409	rs10756038	rs4561528	rs58542926	rs7029757	rs112875651
Effect allele		C	T	A	G	T	C	A	G	T	A	G	G	G	T	T	G	G
β [b]		0.13	0.04	0.04	0.04	0.07	0.05	0.06	0.04	0.03	0.05	0.13	0.24	0.05	0.03	0.17	0.06	0.03
**GEO #**	**CIRM ID**																	
**Control 1**	CW1001	CC	TT	AA	TT	CC	CT	TT	AG	TT	AA	CC	GG	GA	TC	CC	GG	GG
**Control 2**	CW1002	TT	CT	AA	GT	CT	TT	TT	GG	CC	AA	CC	GG	AA	TT	CC	GG	GG
**Control 3**	CW10190	CC	TT	AA	TT	CT	TT	TT	AG	CC	AA	CC	CG	GA	TC	CC	GG	GA
**Control 4**	CW10192	CC	TT	AA	GT	CT	CT	TT	GG	TT	AA	CC	GG	GG	TT	CC	GG	GG
**Control 5**	CW10206	CC	TT	AA	GT	CT	TT	TT	GG	CC	AT	CC	GG	GA	TT	CC	GA	GA
**Control 6**	CW10173	CC	TT	AG	TT	CT	TT	TT	GG	CC	AA	CC	CC	GG	CC	CC	GG	AA
**Control 7**	CW10030	CC	CT	AA	GG	CT	CT	TT	GG	CC	AT	CC	CC	GG	TT	CC	GG	GG
**Control 8**	CW10149	CT	TT	AA	TT	CT	TT	TT	AG	TT	AT	CC	CC	GA	TC	CC	GG	GA
**Control 9**	CW10178	CC	CT	AA	GT	CT	CT	TT	AG	CC	TT	CC	CG	GG	TC	CC	GA	AA
**Control 10**	CW10037	CC	TT	AA	GT	CT	CT	TT	GG	TT	AA	CC	GG	GG	CC	CT	GG	GG
**MASLD 1**	CW10022	CC	CT	AA	TT	CT	TT	TT	AA	CC	AT	CC	GG	GA	CC	CC	GG	AA
**MASLD 2**	CW10182	CC	TT	AA	GT	CT	TT	TT	GG	TT	TT	CC	CG	AA	TT	CC	GG	GA
**MASLD 3**	CW10194	CC	TT	AA	TT	CT	CT	TT	GG	CC	AT	CC	GG	GA	TC	CT	GG	GG
**MASLD 4**	CW10172	CC	TT	AA	TT	CT	CT	AT	GG	TT	AT	CC	GG	GG	CC	CC	GG	AA
**MASLD 5**	CW10166	CC	TT	AA	TT	CT	CT	AT	GG	CC	AA	CC	GG	GG	TC	CC	GG	GA
**MASLD 6**	CW10131	CC	TT	AA	GG	CT	TT	TT	GG	CC	AA	CC	CG	AA	TC	CC	GG	GG
**MASLD 7**	CW10201	CC	TT	AA	GT	CT	TT	TT	GG	TT	AA	CC	GG	GG	TT	CT	GG	GA
**MASLD 8**	CW10165	CC	TT	AA	TT	CC	TT	TT	AG	CC	AA	CC	GG	GG	CC	CT	GG	GG
**MASLD 9**	CW1077	CC	TT	AA	TT	TT	CT	AT	GG	TT	AA	CC	GG	GG	TC	CC	GG	GG
**MASLD 10**	CW10208	CT	CT	AA	GT	CT	CT	AT	AG	TT	AA	CC	GG	AA	TT	CT	GG	GG

[a] rsIDs for MBOAT7 and PNPLA3 are different from those reported by Chen et al;[4] see Table S2.
[a] Effect sizes are those reported by Chen et al[4] for all ancestries.

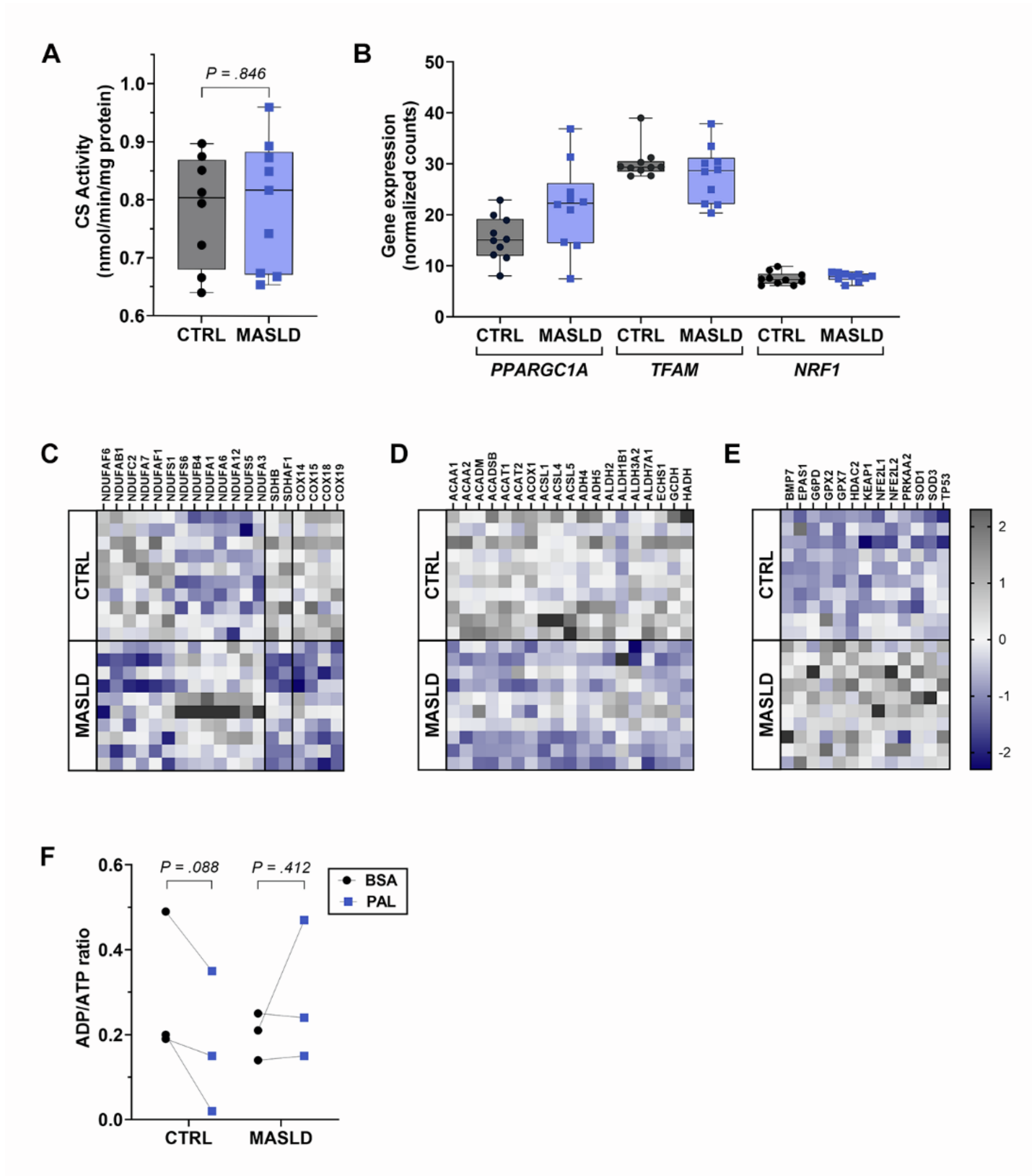

Figure S1. Assays of mitochondrial integrity, displays of differential gene expression, and measurement of ATP synthesis in MASLD vs. control iPSC-Heps.

(A) Citrate synthase (CS) activity in MASLD and CTRL iPSC-Heps. **(B)** Expression of 3 genes involved in mitochondrial biogenesis, measured in MASLD and CTRL iPSC-Heps at day 21 of differentiation. *PPARGC1A*, peroxisome proliferator-activated receptor gamma coactivator 1-alpha; *TFAM*, transcription factor A, mitochondrial; *NRF1*, nuclear respiratory factor 1. **(C-E)** Heatmaps illustrating genes involved in **(C)** oxidative phosphorylation, **(D)** fatty acid oxidation and **(E)** responses to oxidative stress that are differentially expressed ($P < .05$) in MASLD vs. CTRL iPSC-Heps. **(F)** ADP/ATP ratio in MASLD and control iPSC-Heps following treatment with BSA or 700 µM palmitate (PAL) as indicator of fatty acid-induced ATP synthesis. *P* values represent the results of paired t-tests.

REFERENCES

1. Yu J, Chau KF, Vodyanik MA, Jiang J, Jiang Y. Efficient feeder-free episomal reprogramming with small molecules. PLoS One 2011;6:e17557.
2. Si-Tayeb K, Noto FK, Nagaoka M, Li J, Battle MA, Duris C, North PE, Dalton S, Duncan SA. Highly efficient generation of human hepatocyte-like cells from induced pluripotent stem cells. Hepatology 2010;51:297–305.
3. Peaslee C, Esteva-Font C, Su T, Munoz-Howell A, Duwaerts CC, Liu Z, Rao S, Liu K, Medina M, Sneddon JB, Maher JJ, Mattis AN. Doxycycline Significantly Enhances Induction of Induced Pluripotent Stem Cells to Endoderm by Enhancing Survival Through Protein Kinase B Phosphorylation. Hepatology 2021;74:2102–2117.
4. Chen Y, Du X, Kuppa A, Feitosa MF, Bielak LF, O'Connell JR, Musani SK, Guo X, Kahali B, Chen VL, Smith AV, Ryan KA, Eirksdottir G, Allison MA, Bowden DW, Budoff MJ, Carr JJ, Chen YI, Taylor KD, Oliveri A, Correa A, Crudup BF, Kardia SLR, Mosley TH, Jr., Norris JM, Terry JG, Rotter JI, Wagenknecht LE, Halligan BD, Young KA, Hokanson JE, Washko GR, Gudnason V, Province MA, Peyser PA, Palmer ND, Speliotes EK. Genome-wide association meta-analysis identifies 17 loci associated with nonalcoholic fatty liver disease. Nat Genet 2023;55:1640–1650.
5. Duwaerts CC, Le Guillou D, Her CL, Phillips NJ, Willenbring H, Mattis AN, Maher JJ. Induced Pluripotent Stem Cell-derived Hepatocytes From Patients With Nonalcoholic Fatty Liver Disease Display a Disease-specific Gene Expression Profile. Gastroenterology 2021;160:2591–2594 e6.
6. Robinson MD, McCarthy DJ, Smyth GK. edgeR: a Bioconductor package for differential expression analysis of digital gene expression data. Bioinformatics 2010;26:139–40.
7. Kamalian L, Douglas O, Jolly CE, Snoeys J, Simic D, Monshouwer M, Williams DP, Park BK, Chadwick AE. Acute Metabolic Switch Assay Using Glucose/Galactose Medium in HepaRG Cells to Detect Mitochondrial Toxicity. Curr Protoc Toxicol 2019;80:e76.

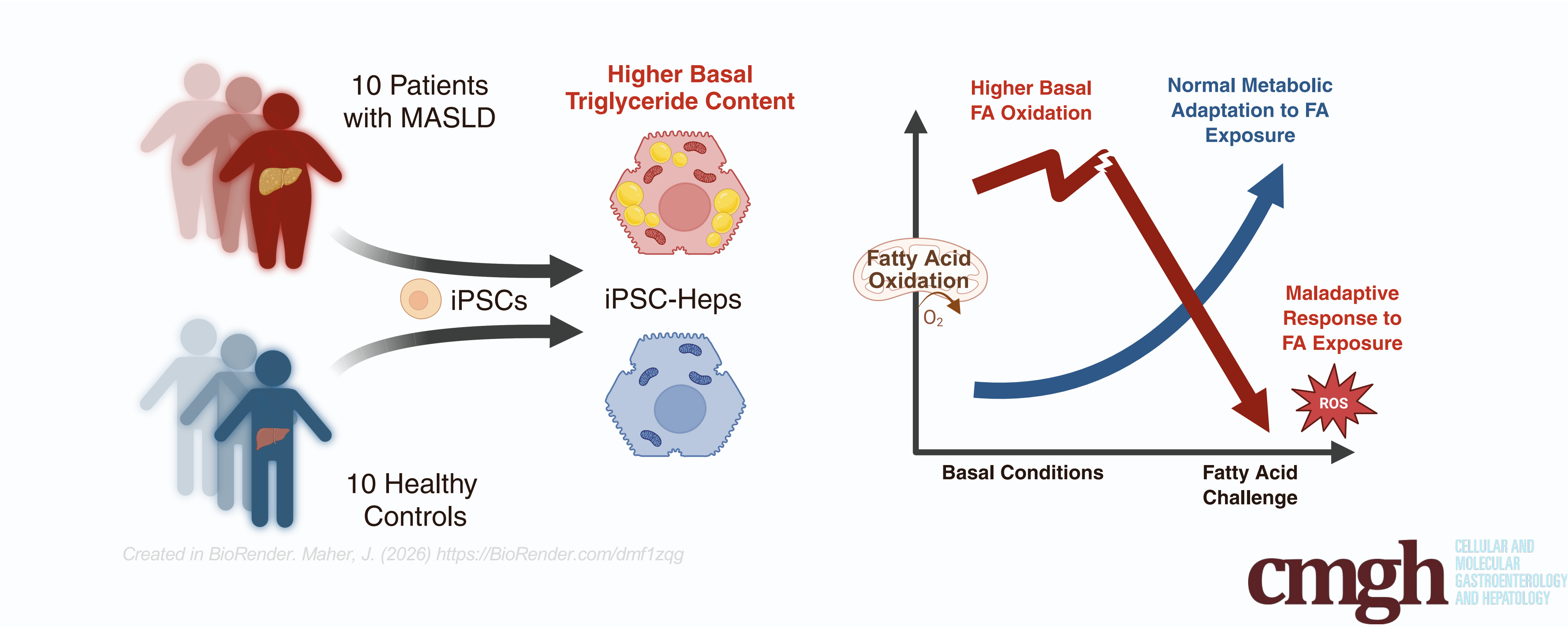
10 Patients with MASLD
Higher Basal Triglyceride Content
iPSCs
iPSC-Heps
10 Healthy Controls
Higher Basal FA Oxidation
Normal Metabolic Adaptation to FA Exposure
Fatty Acid Oxidation
O_2
Maladaptive Response to FA Exposure
ROS
Basal Conditions
Fatty Acid Challenge
Created in BioRender. Maher, J. (2026) https://BioRender.com/dmf1zqg
cmgh
CELLULAR AND MOLECULAR GASTROENTEROLOGY AND HEPATOLOGY
